# Assessment of Bioavailability and Related Bioactivity of Hydroxycinnamic Acids

**DOI:** 10.3390/cimb48070656

**Published:** 2026-06-25

**Authors:** Elica Valkova, Vasil Atanasov, Kiril Kirilov, Kristian Yakimov, Yordan Kutsarov

**Affiliations:** Department of Biological Sciences, Faculty of Agriculture, Trakia University, 6000 Stara Zagora, Bulgaria; vka@mail.bg (V.A.); k.kirilov1973@abv.bg (K.K.); krisss68@mail.bg (K.Y.); kalimok@gmail.com (Y.K.)

**Keywords:** p-coumaric acid, caffeic acid, ferulic acid, bioavailability and activity, sublingual administration

## Abstract

The aim of the present study was to evaluate the bioavailability and associated bioactivity of p-coumaric (p-COA), caffeic (CA), and ferulic (FA) hydroxycinnamic acids (HCAs) isolated from an aqueous extract of plant material. An aqueous extract is more applicable in practice because this form is the most commonly used for oral administration. The p-COA, CA, and FA acids were evaluated for their behavior in the processes of absorption, distribution, metabolism, and excretion (ADME) using modern methods for assessing their functional groups according to Lipinski’s Rule of Five and the Rule of Nines. Given the available data on extensive metabolism of hydroxycinnamic acids during the first pass through the liver, it is necessary to consider an alternative route of administration, namely the sublingual route. Sublingual delivery of exogenous molecules obtained from plant material by extraction may represent a preferable alternative to oral administration, as first-pass hepatic metabolism is bypassed when dosage forms are administered sublingually.

## 1. Introduction

**Figure 1:**
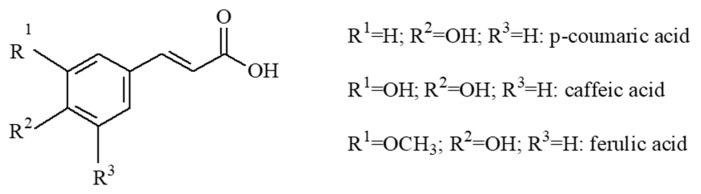
Structure of HCAs.

Hydroxycinnamic acids (HCAs) are a class of naturally occurring phenolic compounds. HCAs are derived from cinnamic acid through hydroxylation, methylation, or esterification. Their traditional medicinal use in the treatment of various diseases has been supported by scientific evidence demonstrating a range of pharmacological effects [1]. These include hypoglycemic [2], hypolipidemic [3], anti-inflammatory [4,5], and antidiabetic activities [6], as well as effects on gastrointestinal motility [7]. Beneficial effects have also been reported in the management of urinary tract infections [8,9,10,11]. The best known and most extensively studied representatives of this class are caffeic acid (CA), para (p-) coumaric acid (p-COA), and ferulic acid (FA) (Figure 1).

However, detailed studies of their biochemical behavior remain limited.

In this review, we discuss and summarize the current body of knowledge regarding these biologically active molecules, which are widely distributed in foods consumed worldwide, including coffee, edible herbs, red wine, whole grains and *Agropyron repens* parasitic plants. These compounds have been associated with improvements in the health and quality of life of both humans and animals. Nevertheless, their beneficial effects following oral administration depend largely on their bioavailability in the body.

In oral administration, a substance is taken through the oral cavity. Absorbed through the gastrointestinal tract, the exogenous molecule enters the mesenteric circulation, which drains into the portal vein. Consequently, before reaching the systemic circulation, the molecule passes through the liver, where it is metabolized. The sublingual route of administration of exogenous molecules is also an oral route; however, unlike conventional oral administration, the substance reaches the stomach either minimally or not at all and is rapidly absorbed through the sublingual mucosa. As a result, the substance enters the systemic circulation directly without passing through the liver, thereby avoiding first-pass metabolism.

In this review, we justify the administration of hydroxycinnamic acids by the sublingual method, which is innovative because it has not been applied previously. The literature contains data on the application of this method to other benzoic acids (salicylic acid) but not to caffeic acid (CA), para (p-) coumaric acid (p-COA), and ferulic acid (FA).

In this context, the present review examines studies related to the solubility, stability, and oral absorption, distribution, metabolism, and elimination (ADME) processes of HCAs. Where appropriate, the data are organized according to the factor evaluated and the method used, thereby providing a straightforward assessment that may serve as a guide for future studies targeting the oral administration of HCAs.

## 2. Characteristics and Properties of Hydroxycinnamic Acids (HCAs)

According to medicinal chemistry principles, functional groups confer specific properties that enable the active ingredients of exogenous molecules to exhibit their desired pharmacodynamic and pharmacokinetic effects.

### 2.1. Definition of Electron-Donating Groups

The electronic effect of a functional group is determined by its ability to donate electrons to neighboring atoms or functional groups or to withdraw electrons from them.

Two main characteristics contribute to the overall electronic effect of a functional group: its ability to participate in resonance and its individual inductive effects.

**Figure 2:**
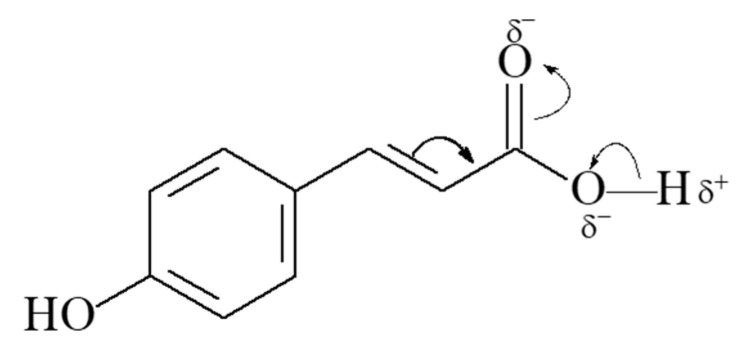
Resonance Structure of p-Coumaric Acid (p-COA).

Resonance occurs when electrons are shared among a group of atoms that contain adjacent double bonds and unshared electron pairs. Since the electrons are equally shared, the overall structure is a hybrid composed of all possible resonance structures. As observed in the case of HCAs, resonance structures can exist within the carboxyl group. Consequently, the negative charge is evenly distributed between the two oxygen atoms. This ability to distribute the positive or negative charge between a group of atoms in the molecule is particularly important because it leads to an increase in the acidity or basicity of specific functional groups. This, in turn, directly affects the degree of solubility of the entire molecule and its transport across membranes (Figure 2) [12,13].

### 2.2. Water–Lipid Solubility

The overall water and/or lipid solubility of an exogenous molecule influences its route of administration, distribution, metabolism, duration of action, and elimination pathway. In general, solubility is determined by the combined contributions of the functional groups present in the molecular structure.

Similar to electronic effects, the contribution of a specific functional group to the overall solubility of a molecule may vary depending on the neighboring groups. The HCAs examined in this review exhibited water solubility greater than 0.1 mg/mL, allowing them to be classified as water-soluble compounds according to the Horter & Dressmann scale [14].

### 2.3. Hydrophilic Functional Groups

Functional groups that increase the water solubility of an exogenous molecule are referred to as hydrophilic. They are characterized by two main properties:

o ionization ability
o hydrogen-bonding ability

To clarify their influence, each of these properties should be considered separately. Acidic and basic groups are capable of ionization and can acquire a negative or positive charge, respectively.

The ionization of a functional group increases the water solubility of the entire molecule because it enables ion-dipole interactions with water. Therefore, the correct identification of acidic and basic groups is essential. A hydrogen bond is a specific type of bond that results from the interaction between two dipoles (i.e., a dipole-dipole interaction) and occurs when a hydrogen atom serves as a bridge between two electronegative atoms [1,2].

A water molecule can act as both a hydrogen-bond donor and a hydrogen-bond acceptor. Thus, groups capable of forming hydrogen bonds can also interact with water through hydrogen bonding, thereby increasing the overall solubility of the molecule in an aqueous environment.

Hydrogen bonds are very important for the interaction between a biologically active molecule and its biological target.

Most current research focuses on the discovery and synthesis of biologically active compounds and on investigating their activity, efficacy, and potential toxicity to humans and the environment. To save time and resources, various mathematical models can be applied before the synthesis of a new bioactive compound or prior to its biological screening in order to establish qualitative and quantitative relationships between its structure and its physicochemical and biological behavior [15].

Molecular descriptors are most commonly used to predict the potential of a given compound as a bioactive substance. In practice, the lipophilicity of the molecule is used in predictive models.

### 2.4. Hydrophobic Functional Groups

According to IUPAC, lipophilicity is the affinity of a molecule or its parts for a lipophilic environment. In addition to lipophilicity, rules for good bioavailability have been established and applied, along with related theoretical approaches for assessing the presence of biological activity of molecules.

The lipophilicity of molecular structures is directly related to transcellular passive diffusion. This process is influenced by the physicochemical properties of the molecule, including molecular weight (MW), partition coefficient (Log *P*), number of hydrogen- bond donors (HBD), and number of hydrogen-bond acceptors (HBA).

When certain functional groups increase the lipid solubility of an exogenous molecule, they are defined as hydrophobic or lipophilic. A characteristic quality they possess is their inability to ionize or form hydrogen bonds, which tends to positively affect the lipid solubility of a biological molecule [16].

The most common lipophilic functional groups include unsubstituted aromatic rings, alkyl groups (also known as aliphatic side chains), saturated carbon rings (also known as alicyclic rings), and halogens.

Lipophilicity can be defined by considering the solute distribution in liquid-liquid or solid-liquid biphasic systems. The traditional method proposed by Hansch and co-workers involves a shake-flask procedure to determine the partition coefficient of the target compound between *n*-octanol and water (Log *P*). While this method is rarely used nowadays, the universal Log *P* scale is still widely applied to represent the lipophilic nature of the molecule of interest.

Theoretically, Log *P* calculations are based on a set of predefined fragments in the Swiss ADME Drug Design Log *P* calculator. This set is based on the dataset by Viswanadhan et al. [16]. where each fragment has a unique name and value. The Log *P* value of a molecule is the sum of the values of its constituent fragments. Thus, the calculated Log *P* values for our three HCAs are as follows:

0.93 (CA) < 1.25 (FA) < 1.43 (p-COA)

This ranking reflects both the lipophilicity and hydrophilicity orders. Specifically, CA has the highest hydrophilicity but the lowest lipophilicity compared to the other two HCAs.

### 2.5. Lipinski’s Rule of Five

Once inside the body, the fate of biologically active molecules and compounds is determined by their absorption, distribution, metabolism, excretion, and toxicity (ADMET) properties. Evaluating and optimizing the action and effectiveness of a given bioactive compound requires a thorough understanding of its pharmacokinetics. Since most bioactive substances of natural origin are not administered intravenously (due to limitations associated with the stability of plant molecules during sterilization and apyrogenation of their solutions), the pharmacokinetic predictor that can indicate the level of intestinal absorption is the effective permeability in the human small intestine. Molecules with higher lipophilicity exhibit better permeability through the phospholipid bilayer of enterocytes; consequently, the level of permeability is directly determined by the lipophilicity of the molecules [16].

Chris Lipinski and his colleagues at Pfizer developed one of the simplest and most commonly used approaches to estimate the likely bioavailability of molecules and compounds.

According to Lipinski et al. [15], for a molecule to be considered highly bioavailable, which is a prerequisite for bioactivity, it must meet certain conditions to be viable as a drug for treatment.

Less absorption and/or insufficient penetration is most likely if:

➢ Molar mass (MW) < 500 Da;
➢ Number of H-bond acceptors (HBA) < 10, (in short as the sum of all O and N atoms);
➢ Number of H-bond donors (HBD) < 5, (in short as the sum of all O-H and N-H bonds);
➢ (Partition coefficient in the n-octanol/water system) Log *P* < 5 (or Mlog *P* < 4.15);

We apply this rule to p-coumaric (p-COA), caffeic (CA), and ferulic (FA) acids (Table 1).

Since Lipinski’s rules are de facto negative, i.e., if a molecule has more than 500 Da molecular mass, then −1 point is recorded for it. This leads to some inaccuracy because a compound with a molecular mass of 499 Da will not receive a negative, while another with 501 Da will be rejected. A difference of 2 DA or 0.004% in molecular masses should not be significant when making a theoretical assessment.

To improve the classification of bioavailability, this factor can be assessed by determining ratios, each of which is formed as a ratio of the eigenvalue of the respective indicator to the limit value according to Lipinski’s rule. For example, given a molecular mass for CA of 180 Da and a correspondingly set limit of 500 Da, the calculated ratio is 0.36. In other words, the molecular mass of CA constitutes 36% of the limit (Equation (1)).

180 Da/500 Da = 0.36 · 100 = 36%(1)

The values for p-COA and FA are calculated using the above formula (Table 2).

In the final column (S), the values for all HCAs are summed sequentially, allowing the molecules to be ranked according to their theoretically calculated bioavailability potential:

CA < FA < p-COA.

Based on these values, p-COA exhibits the highest potential for bioavailability, whereas CA demonstrates the lowest (Table 2). However, because the difference in the ratios among the three HCAs is very marginal, it is necessary to include an additional rule to precisely determine bioavailability.

### 2.6. Rule of Nines

The ionization of functional molecules is a critical factor governing their diffusion through biological membranes. A suitable method for evaluating this property is the Rule of Nines. This technique provides a quick and easy means of calculating the ionization percentage of acidic and basic functional groups, without directly employing the Henderson–Hasselbalch equation.

pH = pKa + log ([A-]/[HA])

By using the absolute value of the difference between the pH of the medium and the pKa of the molecule (or its functional group), the following relationships, known as the Rule of Nines, can be established.

a. If |pH-pKa| = 0, then there is a 50:50 ratio between ionized and unionized forms.
b. If |pH-pKa| = 1, then there is a 90:10 ratio between ionized and unionized forms.
c. If |pH-pKa| = 2, then there is a 99:1 ratio between ionized and unionized forms.
d. If |pH-pKa| = 3, then there is a 99.9:0.1 ratio between ionized and unionized forms.
e. If |pH-pKa| = 4, then there is a 99.99:0.01 ratio between ionized and unionized forms.

It should be noted that the Rule of Nines cannot determine the degree of ionization of a given functional group. It calculates the ratio between these two forms.

When applying the Rule of Nines for CA (pKa = 4.37) and p-SOA (pKa = 4.65):

at pH = 2, corresponding to that in the stomach

- for CA pH-pKa = −2.37
- for p-COA pH-pKa = −2.65

at physiological pH = 7, namely that of lymph and blood

- for CA pH-pKa = 3.37
- for p-COA pH-pKa = 3.65

As is evident from the above calculations, CA and p-COA are exclusively unionized in the stomach (the values are negative) and highly ionized in the blood, respectively.

This suggests good absorption in acidic medium of the non-ionized with the characteristic behavior of lipophilic molecules of CA and p-CoA and good distribution of the same in the blood and lymph flow, where they already exhibit hydrophilic behavior, i.e., are ionized. This conclusion also correlates with Lipinski’s rule, which shows that hydroxycinnamic acids have approximately equal amounts of electron donors and acceptors and, therefore, tend to exhibit both hydrophilic and lipophilic properties, but at the appropriate pH of the medium. At pH values lower than the pKa, HCAs behave as lipophilic molecules, while at higher pH values they acquire a hydrophilic character. This is due to their higher dissociation in this medium [15].

HCAs reach peak plasma concentrations in the bloodstream rapidly, typically within 10 min to 1 h after administration.

These properties influence their probable bioavailability, which forms the basis for their bioactivity. However, to predict the bioactivity of an exogenous molecule accurately, additional factors must also be considered.

## 3. Additional Factors Influencing Biological Activity Besides the Structure of the Compounds

### 3.1. Distribution of Hydroxycinnamic Acids in Mammals and Humans

The distribution process is a typical pharmacokinetic reversible process that varies in speed and extent. It depends primarily on the perfusion of the tissues involved and on the physicochemical properties of the exogenous molecule, which determine its ability to pass through capillary walls. Consequently, the highest concentrations are expected in organs with a rich blood supply.

Studies analyzing the absorption rate in tissues from different animal species and intestinal segments have yielded inconsistent results. For instance, CA shows greater absorption in the porcine ileum, whereas the highest absorption rate in rats occurs within the jejunum [15].

The specific tissues reached by HCAs are the kidneys, liver, muscles, lungs, heart, spleen, and testes. The kidneys (3.2%) and liver (0.3%) are characterized by the highest concentration of HCAs (3.2% and 0.3%, respectively). In the remaining organs, the amount is about 0.1% [15]. Do not be impressed by the low concentration of HCAs, expressed as a percentage, in the well-blooded organs mentioned above. This practically proven concentration also has a very good theoretical explanation.

The perfusion physiological pharmacokinetic model of the distribution of an exogenous molecule in the body assumes the establishment of a rapid equilibrium between the process of distribution between the arterial flow and the tissues and the reverse distribution from the tissues to the venous blood flow. The high rate of these processes determines the fact that the membranes of tissue cells, in this case, do not play a significant barrier role. The distribution process is assumed to be first-order, with a corresponding rate constant. Initially, distribution depends on the concentration of the exogenous molecule in the arterial blood flow, which decreases with time due to the distribution and elimination processes [15].

When the distribution phase is over, the concentration in the tissue will be in equilibrium with the concentration of the exogenous molecule in the venous blood.

The diffusion physiological pharmacokinetic model describes the dependence of the diffusion process on membrane permeability and on the physicochemical characteristics of the exogenous molecule. Here, the affinity of the exogenous molecule for a given tissue or organ is taken into account, while the volume and diffusivity of the tissue are also considered [13].

These theoretical pharmacokinetic models show that only an apparent equilibrium is reached in the arterial blood-tissue-venous blood system. In practice, there is no way for the concentration of exogenous molecules in the arterial blood to become zero in real time because, on the “other” side, the tissue will strive to reach equilibrium with the venous blood flow. Consequently, exogenous molecules will re-enter the arterial flow, since they are part of a vascularly connected system. All ADME processes must be taken into account here and, as will be seen, the concentrations of exogenous molecules in the blood flow will decrease over time.

### 3.2. Metabolism

Microbial metabolism in the intestinal tract significantly influences the fraction available for absorption when exogenous substances are administered orally. The addition of caffeic acid to human fecal microbiota resulted in complete hydrolysis to 3,4-dihydroxyphenyllactate after 4 h [17,18,19,20,21,22,23,24,25,26].

Metabolic transformations occurring in the liver can be divided into two main groups: phase I and phase II.

In phase I of metabolism, the functional groups present in the biomolecule are modified. There are three types of transformations: oxidation, reduction, and hydrolysis. Oxidation is the most common and, in general, these transformations increase the water solubility of the exogenous molecules; however, this increase is not always sufficient to facilitate its elimination from the body.

All phase II metabolic transformations involve the addition of endogenous substances to the exogenous molecule. This process is known as conjugation. It involves the addition of glucuronic acid, sulfate, or the amino acids glycine or glutamine to functional groups that are either originally present in the molecule undergoing transformation or have been added by one or more phase I processes. These conjugated products exhibit increased water solubility, as well as further render the molecule less biologically inactive, after which it is, in most cases, readily excreted.

The metabolism of HCA in mammals proceeds mainly through oxidation, hydrolysis, and conjugation [17,18,19,20,21,22,23,24,25,26]. A dramatic decrease in the maximum concentration (Cmax) measured in the abdominal artery, compared to the portal vein, was observed for CA and p-CoA acids and was accompanied by a corresponding increase in their metabolites (as conjugates), indicating ongoing active metabolism in the liver. The ratio of conjugated to free forms was 3:2, indicating that more than 60% of CA and p-CoA acids in the bloodstream are metabolized, whereas their hydrolysis occurs to a lesser extent [22,23,24].

**Figure 3:**
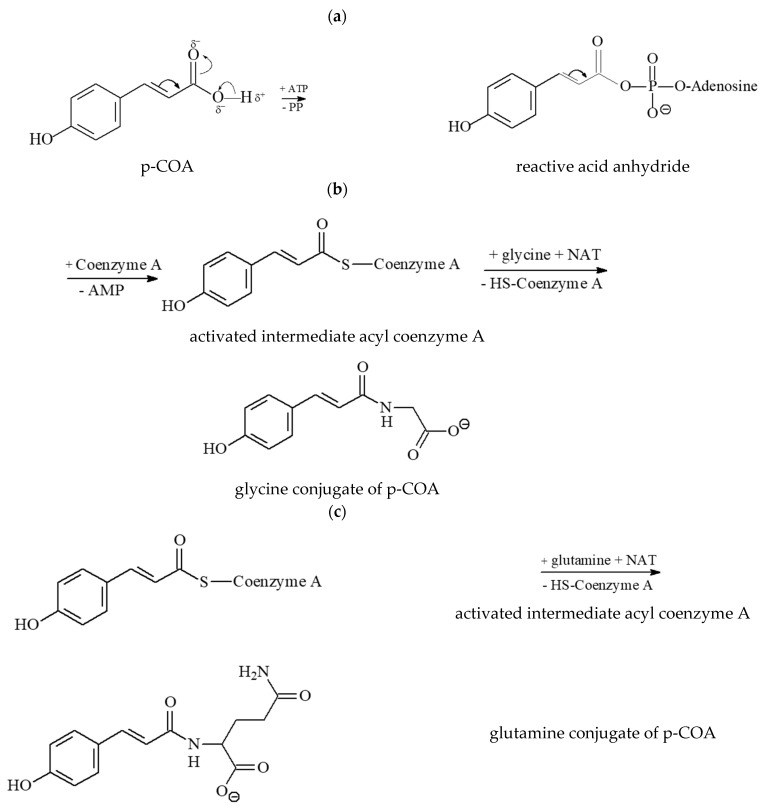
Steps of the mechanism of conjugation of p-COA and the amino acids glycine and glutamine (**a**). First step: p-CoA reacts with adenosine triphosphate (ATP) to form a reactive acid anhydride; (**b**). Second step: Coenzyme A displaces adenosine monophosphate (AMP) to form the activated intermediate acyl coenzyme A, which reacts with glycine to release coenzyme A; (**c**) Third step: Conjugation of the activated intermediate acyl coenzyme A and glutamine to release coenzyme A.

Amino acids can be conjugated to the carboxyl groups of hydroxycinnamic acids, which are initially present in the composition of the exogenous molecule or are introduced during phase I metabolism. The two most commonly used amino acids in this process are glycine and glutamine. Initially, a two-step activation in the appropriate sequence is required before the amino acid can be added to the carboxyl group. An important difference in this metabolic transformation is that the exogenous carboxylic acid is activated instead of the endogenous amino acid [23]. The mechanism of this process is shown in (Figure 3), using p-COA as an example. In the first step, p-COA reacts with adenosine triphosphate (ATP) to form a reactive acid anhydride. In the second step, coenzyme A displaces adenosine monophosphate (AMP) to form the activated acyl coenzyme A intermediate. This is the main function of coenzyme A in biochemical pathways, namely, the activation and transfer of acyl groups. In this metabolic pathway, coenzyme A serves as an active carrier of acyl groups onto p-COA molecules [25]. The final step of the conjugation process is catalyzed by glycine-N-acyltransferase or glutamine-N-acyltransferase. When the aqueous solubility of p-COA is compared to that of its glycine conjugate, it is found that both conjugate molecules contain a carboxyl group; however, the amino acid conjugate provides an additional hydrogen for bonding due to the presence of an amide bond. In the case where glutamine is the amino acid conjugate, it can participate in additional hydrogen-bond formation that is not possible with glycine [23,24,25,26].

Thus, the proposed catalysis by glycine-N-acyltransferase or glutamine-N-acyltransferase of glycine and glutamine proceeds via a more complex three-step catalytic mechanism, in which the enzymes serve as the main catalyst for nucleophilic attack. The amino group of glycine and glutamine must be deprotonated by glycine-N-acyltransferase and glutamine-N-acyltransferase, respectively. A tetrahedral intermediate is formed after nucleophilic attack by the amino group of the amino acid on the thioester carbonyl group. Finally, the tetrahedral intermediate dissociates, forming the final conjugate and releasing CoASH. It should be noted that glycine NAT and glutamine NAT are the enzyme systems that form enzyme–substrate complexes.

### 3.3. Elimination

The elimination of the exogenous molecule is divided into two main processes: biotransformation, which occurs in the liver, and excretion, which occurs in the kidneys. Biotransformation is the process by which the exogenous molecule is converted into a metabolite, whereas excretion is its removal intact and in free form. Based on the data presented so far, it can be concluded that HCAs have a short elimination time when administered orally, which is consistent with their increased distribution in organs involved in elimination [19,20,24].

Morning excretion rates for free CA, p-COA, and FA acids are higher during the first 6 h of urine collection, reaching up to 3% after 48 h, respectively.

Hydroxycinnamic acids (HCAs) are eliminated mainly by biotransformation followed by elimination in the urine. This conclusion can be drawn from the fact that the concentrations of metabolites detected in urine are higher than their unchanged forms [25].

Considering only the free forms of HCAs, p-COA shows the highest concentration in urine [17,27].

## 4. Sublingual Application of HCAs

The sublingual route of administration provides a 3- to 10-fold better ADME response compared with the oral route and is surpassed in this respect only by intravenous (i.v.) and intramuscular (i.m.) injection of substances [20]. Most of the molecules administered by the sublingual route are absorbed by simple diffusion; the sublingual area acts like litmus paper, readily absorbing the substances. However, it should be noted that not all substances are suitable for this type of administration in the body.

### 4.1. Factors Affecting Sublingual Absorption

#### 4.1.1. Solubility in Salivary Secretion

In addition to a certain degree of lipophilicity, the exogenous molecule must also be hydrophilic, such as buccal fluids. In this sense, biphasic solubility is a necessary condition for good absorption.

#### 4.1.2. pH of Saliva and pKa of the Molecule

Since the average pH of saliva is 6.0, this favors the absorption of molecules through the oral mucosa if their pKa is greater than 2 for acids and less than 10 for bases.

According to the above data on hydroxycinnamic acids, we conclude that they theoretically meet the requirements for good absorption in sublingual administration.

### 4.2. Advantages

1. Rapid onset of action is achieved compared to the oral route.
2. The liver is bypassed, and the exogenous molecule is protected from first-pass metabolism. The influence of the digestive enzymes in the mid-gastrointestinal tract is also eliminated.
3. Improved patient comfort due to the elimination of injection-related pain.
4. A low dose provides high efficacy, as it avoids the first-pass metabolism through the liver, which is a prerequisite for reducing the risk of toxic effects associated with high-dose administration.

The first-pass effect is a pharmacological phenomenon in which a drug undergoes metabolism at a specific site in the body. Overcoming this first-pass effect results in an increased concentration of the active drug upon reaching the systemic circulation or site of action. This effect is often associated with the liver, which is the primary site of drug metabolism. However, it can also occur in the lungs, vascular system, gastrointestinal tract, and other metabolically active tissues of the body [25]. Its activation may be the result of various factors, such as plasma protein concentrations, gastrointestinal motility, and enzymatic activity. The extent to which a patient may experience the first-pass effect varies from patient to patient, and this should also be considered when determining appropriate dosing. If it is significantly pronounced in a given patient, the drug may require administration via a different route or formulation to circumvent or reduce its impact to a varying extent [27].

### 4.3. Disadvantages of the Sublingual Route of Introduction of Exogenous Molecules into the Body

In the case of sublingual administration of hydroxycinnamic acids, in addition to the obvious advantages, there are also a number of objective limitations, such as ionization at the pH of the environment, several requirements for the formulation, and the presence of absorption barriers from an anatomical point of view. For this reason, we should accept this route of introduction into the body as an alternative and not as a universal one. In addition to the objective limitations, there are also subjective ones, some of which are:

1. The patient should not smoke while taking sublingual medications, as smoking causes vasoconstriction. This results in decreased absorption of exogenous molecules.
2. An additional disadvantage is the possible staining of teeth caused by long-term use of exogenous molecules with high acidity.
3. The likelihood of a relatively short-lived action in this way of introduction of exogenous substances must be taken into account, which follows from rapid absorption and, in the case of HCAs, rapid metabolism. This problem would be solved by the application of new systems of introduction into the body, which have the necessary properties of controlled release over a period of a week, even with a single administration.

Buccal and sublingual routes of administration are not suitable for bitter or bad-tasting drugs since, in addition to patient discomfort, such formulations may stimulate excessive saliva production, which increases the risk of swallowing.

While absorption may occur by any of paracellular and transcellular mechanisms studied, passive diffusion of drugs from the salivary aqueous phase through the membranes of oral mucosal cells predominates [23]. Therefore, drugs of intermediate polarity are well absorbed, since excessive lipophilicity limits drug dissolution, just as increased polarity limits diffusion across cell membranes. The degree of ionization is also important: less ionized compounds at salivary pH are better absorbed.

#### 4.3.1. Mechanisms Involved in Oral Mucosal Absorption

##### Passive Diffusion

J.C. postulated that the major mechanism involved in the transfer of a drug across the oral mucosa is described by passive diffusion of the unionized form of the drug, in accordance with the pH-partition hypothesis [28,29]. This was first demonstrated for buccal absorption with a series of amphetamines performed by Beckett and coworkers [30]. In this study, drug transport appeared to be a passive diffusion process, since optical isomers of a drug were absorbed to the same extent; absorption was dependent on the concentration of unionized, lipid-soluble form of the drug; and no difference in the percentage absorption of the drug at any given pH value was observed when the drug was administered alone or in combination with other drugs. Since this initial finding, many studies have demonstrated the passive nature of transfer across the oral mucosa [31,32,33,34,35,36,37]. Under these conditions of passive diffusion, the physicochemical properties of the membrane and the drug dictate the transport rate across the biological membrane.

##### Carrier-Mediated Transport

Although passive diffusion is the major transport mechanism for drug permeation across the buccal mucosa, the absorption of certain nutrients from the oral cavity, as shown by Manning and coworkers, has been demonstrated to involve carrier systems [38].

Therefore, further clarification is required in this area. Absorption studies conducted by Sadooghabasian and coworkers, and Evered and coworkers on various vitamins, including L-ascorbic acid, nicotinic acid, and nicotinamide, have been shown to be dependent on the presence of sodium ions, indicating absorption from the oral cavity by carrier-mediated processes [39,40,41,42,43].

Recent investigations carried out by Utoguchi and coworkers have also indicated the existence of an energy-dependent, carrier- mediated monocarboxylic acid transporter system in primary cultures of rabbit and hamster oral mucosal cells and in hamsters in vivo [44,45,46]. Such carrier-mediated systems may be important in the transport of certain drugs, such as salicylic acid. Kurosaki and coworkers have also shown that cefadroxil, an aminocephalosporin antibiotic, is absorbed in the human oral cavity via a specialized transport mechanism, since its absorption demonstrated saturation phenomena and was inhibited in the presence of another aminocephalosporin, cephalexin. Therefore, evidence is building to suggest that passive diffusion of compounds may not be the only mechanism by which compounds permeate the buccal mucosa [44,45].

There has also been a report by Brayton and coworkers regarding the active transport of antibacterial agents in oral mucosa. In a cell line derived from oral epithelium, the uptake of ciprofloxacin and minocycline was not only saturable and inhibited in the presence of other compounds, but the intracellular levels of both antibiotics were also 8–40-fold higher than the extracellular levels, demonstrating an active transport process [47]. Whether the permeability of these compounds across the entire oral mucosa occurs via an active transport process, however, remains to be determined.

#### 4.3.2. Anatomy of the Sublingual Mucosa and Existing Absorption Barriers

The sublingual epithelium and connective tissue form the sublingual mucosa. The sublingual epithelium is composed of squamous epithelium (stratified non-keratinized cells). The thickness of the sublingual epithelium (8–12 cells) is less than that of the buccal epithelial cells (40–50 cells). The non-keratinized nature allows for easy entry of molecules via the sublingual route. The surface cells of the epithelium contain a membrane-covering material, the secretions of which in the intercellular space exist as a vital barrier to the entry of exogenous molecules through the sublingual area. The basal lamina connects the epithelium to the connective tissue and is 150–500 μm thick and consists of both the lamina propria and the submucosa. The lamina propria of the connective tissue contains large blood vessels, nerves, and collagen fibers, allowing for rapid absorption of molecules from this area. Saliva moistens the sublingual area and also coats the tooth and oral mucosa. The pH of saliva also plays a role in sublingual delivery. The epithelial cells of the mucosa are covered with mucus, an intercellular substance synthesized by the salivary glands in the sublingual area, which is made up of oligosaccharide molecules.

These oligosaccharides provide a net negative charge to the mucus at physiological pH. The sublingual mucosa possesses a large amount of polar lipids, such as cholesterol esters, glycosylceramides, and phospholipids. The high fluidity of the membrane and the increased permeability of water and hydrophilic molecules across the sublingual area result from the polar nature of the sublingual mucosa.

#### 4.3.3. Cellular Mechanism of Absorption of Exogenous Molecules

In a sublingual spray delivery system, the drug is sprayed onto the oral mucosa using spray devices in the sublingual area. Three layers of the mucosal lining offer a facile diffusion process for the entry of molecules, but also limit the permeability of certain molecules. The degree of first-pass metabolism is reduced to 48% with sublingual administration compared to up to 28% with an oral dose. Absorption of a drug through the oral mucosa depends on the lipid solubility, permeability, pH, and molecular weight of the drug. Oral epithelial cells and epidermal cells are capable of absorbing molecules by endocytosis. This mechanism is used throughout the stratified epithelium. Active transport processes also operate in the oral mucosa. Acidic stimulation of the salivary glands, as well as vasodilation, facilitates the absorption of molecules into the systemic circulation.

#### 4.3.4. Biopharmaceutical Considerations for Sublingual Administration

After absorption in the sublingual area, exogenous molecules diffuse into the venous blood, then drain into the common trunk, followed by the internal jugular vein, subclavian vein, and brachiocephalic vein, and then directly enter the superior vena cava. The thickness of the epithelial membrane is 100–200 μm in the sublingual area and is non-keratinized. When the drug is administered via sublingual spray, it can reach the systemic circulation through various mechanisms, such as passive diffusion, active or carrier-mediated transport, and endocytosis. The rate of diffusion depends on the molecular weight and solubility of the drug substance, the concentration gradient, temperature, surface area, and proximity of the molecule to the membrane.

The non-ionized form of the drug can be absorbed by passive diffusion. Some substances can be transported by a carrier-mediated process. Some physical models have emerged to study the absorption of drugs from saliva into the systemic circulation through the lipid bilayer. Hydrophilic drugs may have difficulty in crossing such a lipid mucosal layer, and, on the other hand, well-hydrated connective tissues may be a barrier to hydrophobic molecules. Therefore, the lipophilic and hydrophilic nature of the drug substance is essential during the stages of drug product development. The drug should exhibit hydrophobic properties that will be good enough for the lipid bilayer, but only in a minimal amount due to partitioning problems. Oral absorption of molecules is good at Log *P* values of 1 to 5. At values above 5, the solubility of the exogenous substance in saliva may decrease. Typically, a drug formulated for sublingual delivery should have a lower molecular weight to improve its diffusion.

#### 4.3.5. Sublingual Spray Composition

A sublingual spray consists of two main components: the product concentrate, which includes all the components of the formulation except for the propellants. The product concentrate may include dynamic ingredients or a mixture of dynamic ingredients and other necessary agents, such as penetration enhancers, solvents, antioxidants, flavors, sweeteners, hydrophilic polymers, preservatives, acidulants, and cosolvents.

Propellants are used to expel the active ingredients from the containers. Common propellants are hydrocarbons, chlorofluorocarbons, hydrochlorofluorocarbons, and other compressed gases. Propellants used in the formula should not cause a toxic effect in the system and should not interact with the active and inactive ingredients in the formula.

#### 4.3.6. Active Pharmaceutical Ingredient

The ideal drug for a sublingual spray should possess the following characteristics: water solubility that is adequate to provide the desired dosage in the volume of the formulation; suitable sublingual absorption properties that include no sublingual irritation; rapid onset of action; low dosage, typically below 25–30 mg per dose; no production of toxic sublingual metabolites; and no unpleasant odor or taste associated with the drug.

## 5. Various Types of Excipients Are Used in Sublingual Formulations

The most commonly used excipients are as follows:

### 5.1. Buffers

Sublingual secretions can alter the pH of the administered drug, which in turn affects the concentration of unionized drugs available for absorption. Therefore, adequate buffer capacity must be maintained. Examples include sodium phosphate buffer, sodium citrate buffer, and citric acid buffer [48].

### 5.2. Solubilizers

The water solubility of the drug is always a limitation for sublingual drug delivery in solution. Some of the conventional solvents or co-solvents can be used to improve the solubility of the drug. Some biocompatible solubilizers and stabilizers can be used in combination with some hydrophobic absorption enhancers. Sublingual irritation should be considered [48].

### 5.3. Preservatives

For multidose systems, the use of preservatives is considered an important factor in preventing microbial growth. Commonly used preservatives in the sublingual area include methylparaben, ethylparaben, butylparaben, propylparaben; sodium benzoate, benzoic acid, or a mixture of these parabens with phenyl ethyl alcohol and other preservatives, such as benzalkonium chloride, EDTA, and benzyl alcohol. They are not irritating or harmful to the skin [48].

### 5.4. Antioxidants

Antioxidants are used in the formulation to prevent oxidation in the formulation. Commonly used antioxidants are tocopherol, sodium bisulfite, and sodium metabisulfite. At the same time, it should not affect the absorption of the drug or cause sublingual irritation [48].

### 5.5. Surfactants

To modify the permeability of the sublingual area and, in some special cases of nanotechnology incorporation of a sublingual spray, surfactants may be included in the formulation. An example is Polysorbate [48].

### 5.6. Bioadhesive Polymers

Drug compounds used in the sublingual area can be affected by saliva, by eliminating them from the sublingual area. Therefore, some specific substances called bioadhesive polymers are used to retain the drug substance on the surface of the sublingual area. They are called mucoadhesive polymers. The adhesion forces between the drug and the membranes depend on the nature of the polymer, pH, disease state, and mucin turnover. Sublingual irritation leads to the development of some carriers, which is recommended for safety reasons [48].

### 5.7. Penetration Enhancers

Sublingual penetration enhancers are agents added to formulations to increase the absorption of active pharmaceutical ingredients (APIs) through the highly permeable mucous membranes under the tongue. They improve bioavailability by briefly lowering the barrier properties of the oral epithelium without causing permanent damage.

#### 5.7.1. Sodium Dodecylsulfate (SDS)

It is a widely used anionic surfactant consisting of a hydrophobic tail (C12) linked to a hydrophilic sulfate group. It appears as a white crystalline powder, is quite soluble in water and ethanol, and is often used in the cosmetic and pharmaceutical fields to promote solubility, as well as absorption of actives through epithelial membranes, e.g., skin and gastrointestinal mucosa. The permeation enhancer effects are attributable to the alteration of the ordered state of the extracellular lipids by solubilization [48].

#### 5.7.2. Sodium Dehydrocolate (SDC)

It belongs to the class of biliary salts/acids which are defined as amphipathic ionic biosurfactants with a steroid structure, as they are synthesized in the liver from cholesterol. Thanks to its high biocompatibility, it could be widely used as a permeation enhancer through skin buccal, nasal, lung, and intestinal tissues. Moreover, it exerts chemical and enzymatic stabilization of drugs. The absorption enhancement effect is due to the extraction of membrane proteins, interaction with the lipid component of the membranes and the formation of inverse micelles which reversibly increase the fluidity of the apical and basolateral membranes, thus facilitating the passage of drugs [48].

#### 5.7.3. Transcutol^®^ (T)

It is also known as diethylene glycol monoethyl ether. It is a clear liquid characterized by low viscosity, a stability between pH 4–9, and a pleasant odor. It acts as a permeation enhancer by improving drug solubility inside the membranes (alteration of the partition coefficient) rather than directly increasing the drug diffusivity [48].

#### 5.7.4. Lysine Hydrochloride (LYS)

It is a cationic amino acid that belongs to the twenty essential amino acids and, consequently, it is biocompatible, safe and non-toxic. Its chemical permeation enhancement effect is mainly due to the establishment of ionic interactions with the charged groups of the mucosal membrane, thus increasing the diffusion process. Furthermore, lysine could benefit from the amino acid transporters and consequently direct active substances through the epithelial layers [48].

#### 5.7.5. Urea (U)

It is a biocompatible organic compound that appears as a colorless crystalline solid. Numerous studies have depicted its permeation enhancement ability because of its highly moisturizing power (ability to recall and retain water). Moreover, urea is also able to increase the fluidity of the phospholipid bilayer while maintaining the integrity of the membrane protein domains [48].

#### 5.7.6. Menthol (M)

It belongs to the terpenes, which are reported as permeation promoters obtained from natural sources and are widely regarded as safe by the Food and Drug Administration (FDA). The permeation enhancement power of terpenes is mainly linked to their chemical structure and physicochemical properties. In particular, menthol is able to increase the interaction with non-polar membranes, and is also used as a flavoring agent, thereby improving patient compliance [48].

## 6. Discussion

Oral drugs based on active substances of plant origin are a popular and patient-friendly alternative in pharmaceutical treatment strategies. Although they can be formulated to maximize their bioavailability, host-related factors may modulate bioavailability and contribute to heterogeneity in drug response between patients. Some factors are inherently difficult or impossible to control, such as the length of the gastrointestinal tract and the transit time through it. Other non-modifiable factors, such as genetic background, can be considered in patient management, allowing prediction and potential prevention of treatment response variability or adverse reactions [20]. The microbiota is also emerging as an important component that interacts with ingested drugs in complex ways. Understanding the factors influencing drug bioavailability may improve treatment success and help avoid adverse reactions. Furthermore, the implementation of algorithms integrating multiple factors influencing drug bioavailability offers the potential to improve personalized treatment strategies in clinical settings [25].

Based on previous studies and publications, we have applied a multidisciplinary approach to theoretically assess the bioavailability and associated bioactivity of HCAs [24].

Applying this method to other exogenous molecules will facilitate their selection as candidates for practical applications, ultimately leading to improvements in human health.

The dose-response relationship and mechanisms of action of HCAs’ beneficial effects require further research and testing.

Much progress has been made in developing models for specific ADME properties, and, while their potential utility is limited, there are also drawbacks. However, their applicability to the assessment of exogenous molecules of plant origin, other than chemically synthesized drugs, is either weak or not established [20].

This is a consequence of the fact that ADME models have been developed mainly for the pharmaceutical industry, and the available datasets are selected for drug molecules. If these models are applied to other compounds (e.g., of plant origin), the predictive results may be unreliable and, in many cases, the user will not be able to properly assess this, since the applicability domains are not explicitly limited and, in many cases, the training sets and the prediction algorithm are usually not transparent or confidential [24,25].

On the other hand, our review reveals a number of promising methodologies that can be used as effective tools for predicting factors affecting ADME properties [18,23].

It should be borne in mind, however, that their applicability must be determined on a case-by-case basis by comparing predictive with experimental data regarding exogenous molecules of plant origin. This is facilitated by the rich database provided by phytochemistry, botany, and pharmacognosy [20,25,27].

## 7. Conclusions

A characteristic feature of most molecules extracted from plant materials using conventional extraction methods is the poor predictability of bioavailability and associated bioactivity at low-to-moderate doses when administered orally, despite their good absorption. This is related to extensive hepatic first-pass metabolism, which occurs due to their specific nature and their natural biosynthesis in plants.

The methods applied to assess absorption and, consequently, the bioavailability and bioactivity of HCA, such as Lipinski’s Rule and the Rule of Nines, as well as other physicochemical and pathophysiological approaches, provide promising results for future studies.

When reviewing secondary metabolites of plant origin and exogenous ones in terms of their application in animals and humans, it can be concluded that they possess significant therapeutic potential, as they represent biologically active molecules, but are administered in high concentrations. This, in turn, may pose a risk of exceeding the therapeutic window and inducing toxic effects. In light of the present study and the existing literature, it can be inferred that a new route of administration should be proposed, namely sublingual spray delivery. This is a simple and accessible approach; however, it requires an appropriate dosing mechanism for dose control, inertness of the primary packaging toward the biologically active substance placed in it, and the presence of atmospheric pressure.

In addition, we acknowledge the presence of limitations related to the influence of ionization at the pH of saliva and practical barriers affecting absorption, and these should be addressed through formulation modifications.

Furthermore, we recognize that this review is largely theoretical and is based on the current body of knowledge and the publications available to date, so it seems necessary to conduct practical experiments concerning the sublingual administration of HCAs in the future.

## Figures and Tables

**Table 1:** Application of Lipinski’s rule to p-coumaric (p-COA), caffeic (CA) and ferulic (FA) acids.

HCAs	MW	Log *P*	HBD	HBA
CA	180	0.93	3	4
p-COA	164	1.43	2	3
FA	194	1.25	2	4

The values of the three-hydroxycinnamic acids thus presented were calculated with the help of Swiss ADME Drug Design.

**Table 2:** MW, Log *P*, HBD, HBA, S values for p-CoA, FA and CA acids calculated by Equation (1).

HCAs	MW	Log *P*	HBD	HBA	S
CA	0.36	0.186	0.6	0.4	1.546
p-COA	0.328	0.286	0.4	0.3	1.314
FA	0.388	0.25	0.4	0.4	1.438

## Data Availability

No new data were created or analyzed in this study. Therefore, data sharing is not applicable to this article.

## Abbreviations

ADME	absorption, distribution, metabolism and excretion
HCAs	hydroxycinnamic acids
P-COA	p-coumaric acid
CA	caffeic acid
FA	ferulic acid
